# Udder health in beef cows and its association with calf growth

**DOI:** 10.1186/1751-0147-56-9

**Published:** 2014-01-30

**Authors:** Karin Persson Waller, Ylva Persson, Ann-Kristin Nyman, Lena Stengärde

**Affiliations:** 1 Department of Animal Health and Antimicrobial Strategies, National Veterinary Institute (SVA), SE-751 89 Uppsala, Sweden; 2 Department of Clinical Sciences, Swedish University of Agricultural Sciences, SE-750 07 Uppsala, Sweden; 3 Växa Sverige, Box 288, SE-751 05 Uppsala, Sweden; 4 Swedish Animal Health Service, SE-391 28 Kalmar, Sweden

**Keywords:** Beef cows, Mastitis, Intra-mammary infections, Blind quarters, Risk factors, Calf weaning weight

## Abstract

**Background:**

Studies outside the Nordic countries have indicated that subclinical mastitis (measured by milk somatic cell count or the California Mastitis Test), intramammary infections (IMI), or blind quarters in beef cows may have negative effects on beef calf growth. Knowledge on prevalence of such udder health problems in Swedish beef cows is scarce. Therefore, the main aim of this study was to investigate subclinical mastitis, IMI and udder conformation in a number of beef cow herds. Production of β-lactamase in staphylococci was also investigated. Associations between certain cow factors and subclinical mastitis and IMI, and associations between cow and calf factors and 200 day calf weaning weight were also studied. The herds were visited once within a month after calving and once at weaning. Udder examination and quarter milk sampling, for somatic cell count and bacteriology, were performed in 8 to 12 cows per herd and occasion.

**Results:**

Approximately 50%, 40% and 10% of the cows had subclinical mastitis, IMI, and at least one blind quarter, respectively, but the prevalence varied markedly between herds. Intramammary infections (mainly due to staphylococci) were identified in 13-16% of the milk samples. Less than 5% of the staphylococcal isolates produced β-lactamase. Approximately 11% of the cows sampled twice had the same IMI (mostly *Staphylococcus aureus*) at both samplings. Cow factors of importance for subclinical mastitis and/or IMI were teat and udder shape, breed, parity, presence of blind quarters, and cow hygiene. No significant associations were found between udder health parameters studied and calf weaning weights.

**Conclusions:**

Subclinical mastitis and IMI, but not blind quarters, were common in beef cows, but the prevalence varied markedly between herds. Most IMI were caused by staphylococci and more than 95% of those were sensitive to penicillin. Cows with large funnel-shaped teats or pendulous udder after calving, and cows with blind quarters were at risk of having subclinical mastitis and/or IMI. Poor hygiene was also a risk factor for udder health problems. No significant associations were found between udder health and calf weaning weight. More studies on risk factors are warranted to improve advisory services on awareness and prevention of mastitis in beef cows.

## Background

The main goal in beef cow herds is production of healthy calves. To keep calves healthy and fast growing a good start in life is of utmost importance. The milk production of the cow is considered to be an important factor affecting calf growth before weaning [1]. Factors that reduce milk production may therefore have negative impact on calf weaning weights.

Mastitis, a multifactorial disease often associated with bacterial intramammary infections (IMI), significantly reduces milk production and causes large economical losses in dairy herds both in its clinical and subclinical form. The knowledge on mastitis in beef cows is, however, limited. Most publications have studied subclinical mastitis or IMI, and were performed in the US. Those studies indicate that the prevalence of subclinical mastitis, measured by milk somatic cell count (SCC) or the California Mastitis Test (CMT), or prevalence of IMI may vary markedly between herds [1-11]. As an example, the within-herd cow prevalence of IMI varied between 7% and 66% in those studies. Studies have indicated that udder health is important for growth of beef calves as subclinical mastitis or IMI in the dams have been associated with a 5-12% reduction of calf weaning weight [1-3,8,11]. Some IMI, especially *Staphylococcus aureus*, seem to have more negative effects on calf weaning weights than others [1,8,11]. Cows with one or more blind quarters may also have calves with reduced growth [2].

It is well known that clinical mastitis sometimes occurs in Swedish beef cows, but the prevalence of subclinical mastitis and IMI is not known and neither is the distribution of different udder pathogens. Moreover, the prevalence of blind quarters is also unknown. Therefore, the main aim of the study was to investigate these udder health indicators in a number of beef cow herds. Production of β-lactamase in staphylococci was also investigated. In addition, associations between certain cow factors and subclinical mastitis and IMI, as well as associations between cow and calf factors and the adjusted 200 day calf weaning weight were studied.

## Methods

### Study design

Ten beef cow herds were selected based on the following criteria; the herd should be situated in the southern third of Sweden, be affiliated to the official national beef production scheme (KAP; a Swedish acronym meaning meat, breeding and production), have more than 20 cows, have suitable cattle handling facilities, and be willing to participate in the study.

Each herd was visited twice during 2012. At the first visit, in late winter/spring, cows within one month after calving were sampled. The second visit, in autumn, was performed just before weaning. When possible the same cows were sampled at both visits. All cows were housed at the first visit, and at the second visit they were either on pasture or recently housed after the end of the pasture season. The visits were performed by one of three veterinarians from the Swedish Animal Health Service. At each visit the udders of approximately 10 cows were examined by visual assessment and manual palpation. Special attention was given to presence of teat and udder shape, as described previously [12], and presence of blind quarters. Quarter milk samples for analysis of SCC were taken after the first strips of milk had been discarded. Thereafter, milk samples for bacteriology were taken aseptically according to routines for sampling of dairy cows. When possible CMT (scored 1 to 5, where 1 is healthy and 5 is strong positive reaction) was also performed. The milk samples were cold-stored and sent to the National Veterinary Institute (SVA) for analyses the day after sampling. The cleanliness of the udder, hind legs, and flanks of the cows was scored using a four grade scale where 1 was clean and 4 was very dirty [13]. Herd data (number of cows/year, number of calves/cow/year), cow data (identity, breed, year of birth, parity, calving date, calf identity) and calf data (identity, mother identity, breed, day of birth, single/twin, birth weight, 200 day weaning weight) were collected in association with the second visit.

The study design was approved by the Regional Committee for Ethics in Animal Research in Uppsala, Sweden (application number C363/11).

### Laboratory analyses

The SCC was evaluated using CMT, and the Delaval Cell Counter (DCC). Bacteriological growth was investigated as described previously [14]. Species differentiation of coagulase-negative staphylococci (CNS) was performed using Maldi-TOF [15]. All staphylococcal isolates were tested for β-lactamase production using the clover-leaf method [16].

### Data editing and statistics

Subclinical mastitis in an udder quarter was defined as DCC-SCC ≥200 000/ml. The bacteriological result per quarter was classified as no growth, contamination (≥3 different bacterial species), and IMI with specific udder pathogens (any IMI, any major IMI (defined as all pathogens except CNS and *Corynebacterium bovis*), CNS IMI, *S. aureus* IMI).

The DCC-SCC in quarters with no growth were compared after calving and at weaning using the Student’s t-test. Due to a large number of missing values for CMT from the herd investigation only CMT values from the laboratory investigation were used in the statistical evaluation. Comparisons of CMT-scores between quarters with different bacterial findings were performed using the Chi-square test.

Associations between DCC-SCC and bacterial growth on udder quarter level were evaluated using mixed-effect univariable regression analyses, with cow and herd as random factors. Associations between cow factors (breed, parity, hygiene score (1–4), teat shape (normal/funnel shaped), udder shape (normal/pendulous), number of lactating udder quarters (3/4)) and number of udder quarters with subclinical mastitis (DCC-SCC ≥200 000/ml or CMT 3–5) or IMI (any IMI, any major IMI, CNS IMI, *S. aureus* IMI) per cow at each of the two samplings were evaluated using the Fisher’s exact test.

Associations between the dependent variable calf 200 day weaning weight, and the independent variables subclinical mastitis (number of quarters with DCC-SCC ≥200 000/ml (0, ≥1)) or IMI in one or several quarters per cow (number of quarters with any IMI (0, 1, 2, 3–4, or 0, ≥1), number of quarters with any major IMI (0, ≥1), number of quarters with CNS IMI (0, 1, 2–3, or 0, ≥1), number of quarters with *S. aureus* IMI (0, 1, 2–3, or 0, ≥1) at each sampling occasion), were investigated using mixed-effect univariable and multivariable regression analyses (with cow and herd as random factors). Moreover, calf factors (gender, twin (yes/no), birth weight), general cow factors (breed, parity) and cow health factors (hygiene score, number of lactating quarters, teat and udder shape) were also included as independent variables. For the multivariable models collinearity between the independent variables was assessed pair-wise by calculation of Spearman rank correlations. If there was proof of collinearity (r ≤ 0.70) the variable with lowest *P*-value in the univariable analysis was selected. Moreover, in all the multivariable models biologically plausible two-way interactions between the main effects were tested. The model fit of the multivariable analyses was tested by visual examination of diagnostic plots [17]. All statistical analyses were performed using Stata (Release 11.2; College Station, TX, USA: StataCorp LP).

## Results

The median number of cows per herd year 2012 was 58 (range 15–103). Breeds present were Simmental (4 herds), Hereford (4 herds) and Limousin (2 herds). Two of the Simmental herds and one of the Hereford herds also had cross-bred cows. Eight herds housed the cows in cold loose housing systems during winter while one herd had a tied-up system and one herd had a combination of tied-up and loose housing systems during winter. In all herds the cows were on pasture from mid April/end of May to end of September/end of November.

The number of sampled cows per herd and occasion varied between 8 and 12. A total of 109 individuals were examined of which 83 (76%) participated at both samplings. The distribution of breeds were 37 (34%) Hereford, 34 (31%) Simmental, 21 (19%) Limousin, and 17 (16%) cross-breeds. Most (64%) cows were in their first to third lactation. The cow hygiene score was, on average, 2.1 (SD 0.6) and 1.8 (SD 0.7) at the first and second visit, respectively. The median cow hygiene score was 2 at both occasions.

### Prevalence of subclinical mastitis, IMI and blind quarters

**Figure 1:**
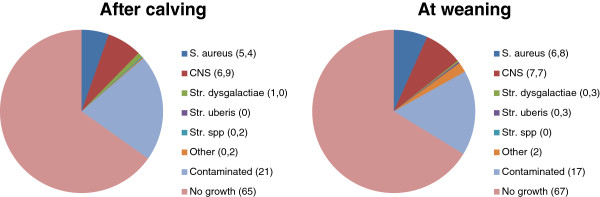
**Bacterial findings in quarter milk samples.** Proportions (%) of udder quarter milk samples from beef cows with different types of bacterial findings within one month after calving (n=403) and at weaning (n=351).

At quarter level, 20 to 30% of the quarters had a DCC-SCC indicating subclinical mastitis, and around 15% of the quarters had IMI, while blind quarters were rather uncommon (Table 1). A total of 332 quarters were sampled twice and 19 (6.1%) of those quarters had subclinical mastitis at both occasions. Overall, IMI was identified in 13% and 16% of the quarters after calving and at weaning, respectively (Figure 1). Most IMI were staphylococci (CNS or *S. aureus*), and the majority of those (94.6% and 95.4%, respectively) did not produce β-lactamase. Seven CNS species (*S. chromogenes, S. haemolyticus, S. hyicus, S. sciuri, S. simulans, S. succinus,* and *S. xylosus*) were identified. The majority (66%) of the CNS isolates was *S. chromogenes,* and the second most common species was *S. sciuri* (18%). The other CNS species each constituted less than 4% of the isolates.

**Table 1:** Prevalence of udder quarters with subclinical mastitis (SCC ≥ 200 000/ml), intramammary infection (IMI) or no milk production (blind quarter) in beef cows investigated within a month after calving and at weaning in 10 beef cow herds

**Variable**	**After calving (n = 104 cows)**		**At weaning (n = 90 cows)**	
	**Total number of investigated quarters**	**Number (%) of affected quarters**	**Total number of investigated quarters**	**Number (%) of affected quarters**
SCC ≥200 000/ml	396	72 (18.2)	351	103 (29.3)
IMI	403	56 (13.9)	351	56 (15.9)
Blind quarter	416	11 (2.6)	358	9 (2.8)

Results on cow level are given in Table 2. Approximately 50% of the cows had subclinical mastitis in at least one udder quarter, while around 40% and 16% of the cows had any IMI or *S. aureus* IMI, respectively. Approximately 11% of the cows sampled twice had *S. aureus* IMI at both samplings. Around 10% of the cows had one blind quarter, and none of the cows had more than one blind quarter. As shown in Table 3, the prevalence of the different outcomes varied markedly among herds.

**Table 2:** **Cow level prevalence of beef cows with at least one udder quarter with subclinical mastitis (SCC ≥200 000/ml), intramammary infection (IMI) of any bacteria, *Staphylococcus aureus *** **IMI*, *** **or no milk production (blind quarter) within a month after calving and at weaning in 10 beef cow herds**

**Variable**	**Cows after calving (n = 104) n (%)**	**Cows at weaning (n = 90) n (%)**
SCC ≥200 000/ml	48 (46.1)	52 (57.8)
Any IMI	42 (40.4)	38 (42.2)
*S. aureus* IMI	15 (14.4)	17 (18.9)
Blind quarter	11 (10.6)	8 (8.9)

**Table 3:** **Herd level prevalence of beef cows with at least one udder quarter with subclinical mastitis (SCC ≥200 000/ml), intramammary infection (IMI) of any bacteria, *Staphylococcus aureus *** **IMI*, *** **or no milk production (blind quarter) within a month after calving and at weaning in 10 beef cow herds**

**Variable**	**After calving**		**At weaning**	
	**Number of herds with ≥1 cow affected**	**Proportion of cows affected per herd median (range)**	**Number of herds with ≥1 cow affected**	**Proportion of cows affected per herd median (range)**
SCC ≥200 000/ml	10	52 (18–67)	9	69 (0–90)
Any IMI	9	38 (0–67)	10	43 (22–60)
*S. aureus* IMI	7	13 (0–33)	9	21 (0–33)
Blind quarter	6	9 (0–40)	4	0 (0–40)

### Comparisons of DCC-SCC after calving and at weaning

The average DCC-SCC in quarters with no growth after calving and at weaning was 147 000/ml (SD 419 000/ml) and 196 000/ml (SD 319 000/ml), respectively, and did not differ significantly (Student’s t-test, *P* = 0.41). The corresponding median SCC was 44 000/ml and 52 000/ml, respectively.

### Associations between DCC-SCC or CMT and bacterial findings

The DCC-SCC varied markedly in quarters with IMI, but was significantly higher in IMI quarters than in quarters with no growth (Table 4). Quarters infected with CNS had lower DCC-SCC than quarters with other IMI. Quarters with *S. chromogenes* had higher SCC (median 271 000/ml) than quarters infected with other CNS (median 70 000/ml; Student’s t-test, *P* = 0.005).

**Table 4:** Associations between somatic cell counts (SCC x1000/ml) and intramammary infection (IMI) in udder quarter milk samples taken from beef cows within a month after calving and at weaning in 10 beef cow herds

**Bacterial findings**	**After calving**			**At weaning**		
	**Median SCC (50% central range)**	**n**	***P*-value**	**Median SCC (50% central range)**	**n**	***P*-value**
IMI						
No	48 (22–104)	314		44 (13–188)	295	
Yes	346 (75–1113)	50	<0.0001	303 (58–1041)	56	<0.0001
IMI, not CNS^a^						
No	48 (22–104)	312		44 (13–188)	295	
Yes	820 (185–2605)	24	<0.0001	370 (75–1946)	29	<0.0001
IMI						
No	48 (22–104)	314		44 (13–188)	295	
CNS^a^	106 (66–425)	26		218 (30–802)	27	
* S. aureus*	820 (185–2487)	20		419 (97–2318)	21	
Other	1782 (353–3208)	4	<0.0001^b^	239 (37–677)	8	<0.0001^c^

^a^CNS = coagulase negative staphylococci.

^b^CNS, *Staphylococcus aureus* and other pathogens (only *Streptococcus dysgalactiae*) had significantly higher SCC than no IMI; *S. aureus* and other pathogens had significantly higher SCC than CNS.

^c^CNS and *S. aureus* had significantly higher SCC than no IMI; *S. aureus* had significantly higher SCC than CNS and other pathogens (2 *Escherichia coli*, 1 *Serratia marcescens*, 1 *Str. dysgalactiae*, 1 *Str. uberis* and 3 coagulase positive staphylococci (not *S. aureus*).

The proportion of udder quarters with CMT 3–5 (indicating sub-clinical mastitis) was significantly (*P* < 0.0001) higher in quarters with IMI (mean score 2.04 (SD 1.22); median score 2) than in quarters with no growth (mean score 1.34 (SD 0.68); median score 1). The proportion of quarters with no growth, any IMI, any major IMI, CNS IMI, and *S. aureus* IMI having CMT 3–5 was 4.3%, 27.3%, 38.9%, 16.1% and 40.5%, respectively.

### Associations between cow factors and prevalence of subclinical mastitis or IMI at cow level

Cow factors studied were breed, parity, hygiene, teat and udder shape, and presence of blind quarters. Factors having significant effects on the outcome variables after calving and at weaning are presented in Tables 5 and 6, respectively. Presence of quarters with subclinical mastitis after calving was more common in cows with large funnel-shaped teats or pendulous udder than in cows with normal teat and udder shape. Presence of any IMI was also more common in cows with large funnel-shaped teats. Hereford and Simmental had the lowest and highest, respectively, proportions of cows with subclinical mastitis after calving. Cows in the first and second lactation had lower prevalence of IMI after calving than older cows. IMI was less common in cows with four lactating quarters than in cows with one blind quarter. Cows with poor hygiene score had more CNS IMI after calving, and more subclinical mastitis and IMI at weaning than clean cows.

**Table 5:** **Cow factors with significant (*P *** **< 0.10) effect on prevalence of subclinical mastitis (SCC ≥200 000/ml) or intramammary infections (IMI) in beef cows examined within one month after calving in 10 beef cow herds**

**Outcome**	**Cow factor**	**Class**	**Number of cows with different numbers of quarters affected (QA)**			**Total numbers *P*-values**
Quarters with SCC ≥200 000/ml			0 QA	1 QA	≥2 QA	
	Breed	Hereford	23	12	0	35
		Cross bred	8	5	4	17
		Limousin	12	3	6	21
		Simmental	12	9	8	29
		*Total*	55	29	18	*P* = 0.008
	Teat shape	Normal	37	12	8	57
		Large funnel-shaped	16	15	10	41
		*Total*	53	27	18	*P* = 0.038
	Udder shape	Normal	48	17	15	80
		Pendulous	7	12	3	22
		*Total*	55	29	18	*P* = 0.012
Quarters with CMT^a^ ≥3			0 QA	1-4 QA		
	Teat shape	Normal	52	5		57
		Large funnel-shaped	27	14		41
		*Total*	79	19		*P* = 0.004
	Udder shape	Normal	68	12		80
		Pendulous	14	8		22
		*Total*	82	20		*P* = 0.035
Quarters with any IMI			0 QA	1 QA	≥2 QA	
	Parity	1	22	6	1	29
		2	13	2	3	18
		3	6	7	3	16
		4	8	7	0	15
		5-6	8	5	3	16
		≥7	4	3	1	8
		*Total*	61	30	11	*P* = 0.083
	Teat shape	Normal	39	13	5	57
		Large funnel-shaped	19	16	6	41
		*Total*	58	29	11	*P* = 0.094
	Blind quarter present	No	58	25	8	91
		Yes	3	5	3	11
		*Total*	61	30	11	*P* = 0.031
						
Quarters with any major IMI			0 QA	1 QA	≥2 QA	
	Blind quarter present	No	77	11	3	91
		Yes	6	3	2	11
		*Total*	83	14	5	*P* = 0.025
Quarters with CNS^b^ IMI			0 QA	≥1 QA		
	Hygiene	1	15	1		16
		2	43	21		64
		3-4	18	3		3
		*Total*	76	25		*P* = 0.048
Quarters with *S. aureus* IMI			0 QA	≥1 QA		
	Blind quarter present	No	80	11		91
		Yes	7	4		11
		*Total*	87	15		*P* = 0.054

^a^CMT = California Mastitis Test.

^b^CNS = coagulase negative staphylococci.

**Table 6:** **Cow factors with significant (*P *** **< 0.10) effect on prevalence of subclinical mastitis (SCC ≥200 000/ml) or intramammary infections (IMI) in beef cows examined at weaning in 10 beef cow herds**

**Outcome**	**Cow factor**	**Class**	**Number of cows with different numbers of quarters affected (QA)**^**a**^			**Total numbers *P*-values**
Quarters with SCC ≥200 000/ml	Hygiene		0 QA	1 QA	≥2 QA	
		1	9	8	11	28
		2	29	13	11	53
		3-4	1	4	4	9
		*Total*	39	25	26	*P* = 0.051
Quarters with CMT^b^ ≥3	Hygiene		0 QA	1-4 QA		
		1	16	10		26
		2	40	8		48
		3-4	5	4		9
		*Total*	61	22		*P* = 0.044
Quarters with any IMI	Hygiene		0 QA	1 QA	≥2 QA	
		1	13	7	8	28
		2	36	12	5	53
		3-4	3	4	2	9
		*Total*	52	23	15	*P* = 0.067
Quarters with major IMI	Blind quarter present		0 QA	1 QA	≥2 QA	
		No	65	13	4	82
		Yes	4	1	3	8
		*Total*	69	14	7	*P* = 0.026
Quarters with *S. aureus* IMI	Blind quarter present		0 QA	≥1 QA		
		No	70	12		82
		Yes	4	4		8
		*Total*	74	16		*P* = 0.031

^a^QA refers to outcome at the same row.

^b^CMT = California Mastitis Test.

### Associations between cow and calf factors, and 200 day calf weaning weight

The multivariable analyses revealed that none of the cow udder health factors had any significant (*P* > 0.05) association with calf weaning weight. Factors that had a significant effect on weaning weight were gender of the calf (*P* < 0.001), weight at birth (*P* = 0.004) and breed (*P* < 0.001). The weaning weight increased with 33 kg if the calf was a male, and with 1.7 kg per kg increasing weight at birth. The weaning weight was 82 kg, 18 kg and 68 kg higher in cross-breeds, Limousin and Simmental, respectively, than in Hereford. The significant factors explained 69% of the variation in calf weaning weight.

## Discussion

The results of this study show that subclinical mastitis and IMI were common in the investigated beef cows. A somewhat higher proportion of cows was affected by subclinical mastitis than by IMI, but the proportions of affected cows varied markedly between herds. The proportions of beef cows with subclinical mastitis or IMI after calving and at weaning were similar. The large variation between herds was in line with studies from other countries [1-11]. According to Newman *et al.*[8] and Duenas *et al.*[2] the prevalence of IMI is highest at the end of lactation, but in the present study no such difference was observed between sampling occasions. In this study, as well as in other studies [2,9], both young and old cows were affected, but the prevalence increased with increasing parity. The most common IMI found were CNS and *S. aureus,* which is in line with previous studies [1,2,4,6,8,9].

Regarding cow factors of importance for the prevalence of subclinical mastitis or IMI the results indicate that large funnel-shaped teats, pendulous udder, presence of blind quarters, and poor hygiene are risk factors for udder health problems. The importance of teat and udder shape is in line with recommendations from the USA [12]. The fact that cows having blind quarters had more problems with subclinical mastitis and IMI in the other udder quarters is likely a result of the spread of an IMI from the blind quarter to the other quarters within the udder. An association between blind quarters and poor udder health was also found in previous studies [2,6]. The results indicate that the risk for udder health problems within a herd can be reduced by culling cows that have large funnel-shaped teats or pendulous udders after calving, and cows with blind quarters. Other risk factors for poor udder health in beef cows that have been suggested are holding cows within a limited space, presence of many flies around the cows, cross-suckling calves, and wet and muddy weather [18], but those factors were not investigated in the present study.

In the present study, a SCC cut-off of 200 000/ml was chosen as the definition of subclinical mastitis. This cut-off is based on studies on dairy cows [19,20], and it is not known if the cut-off is appropriate also for beef cows. Moreover, only one milk sample per quarter was taken at each sampling period for evaluation of IMI, which might increase the risk of false negative results. It was not, however, practically possible to take several samples in this study. The results indicate, however, that the SCC cut-off and the sampling procedure were adequate as udder quarters with no growth mostly had SCC below 200 000/ml, and that the SCC was significantly higher in quarters with IMI. Differences in SCC between quarters with different types of IMI were also as expected based on studies on dairy cows.

In contrast to earlier studies [1-3,8,11] a significant association between subclinical mastitis or IMI, and 200 day calf weaning weight was not observed. However, in line with our results, Paape *et al.*[9] did not find any effect of IMI on calf weaning weight, and Simpson *et al.*[10] found no difference in weaning weights between calves from Simmental cows with low or high SCC. The differences between studies may be due to differences in study design, such as number of herds and animals, breeds included, or statistical methods. It is, for example, likely that the effect of a decreased milk production on calf growth is less pronounced in breeds producing more milk. In this study, gender of calf, breed and weight at birth had substantial influence on the weaning weight. Factors like quality and amounts of feed or pasture may also be important, but this was not investigated. As the number of herds and cows included in the present study was relatively small the results should be interpreted with caution.

## Conclusions

Subclinical mastitis and IMI, but not blind quarters, was common in beef cows both within one month after calving and at weaning, but the prevalence varied markedly among herds. Only around 11% of the cows sampled twice had the same IMI (mainly *S. aureus*), indicating persistent IMI, at both samplings. Most IMI were caused by staphylococci and more than 95% of those were sensitive to penicillin. Cows that had large funnel-shaped teats or pendulous udders after calving, and cows with blind quarters were at risk of having subclinical mastitis and/or IMI. Poor hygiene was also a risk factor for udder health problems. In this study, no significant associations were found between udder health and calf weaning weight. The high prevalence of subclinical mastitis and IMI, and the marked variation in herd prevalence indicate that more studies on risk factors are warranted to improve advisory services on awareness and prevention of mastitis in beef cows.

## Abbreviations

CMT: California mastitis test; DCC-SCC: DeLaval cell counter somatic cell count; IMI: Intramammary infection; SCC: Somatic cell count.

## Competing interests

The authors declare that they have no competing interests.

## Authors’ contributions

KPW conceived of the study, was responsible for its coordination and design, and drafted the manuscript. YP participated in the design and coordination of the study, and helped to draft the manuscript. AN performed the statistical analyses and helped to draft the manuscript. LS participated in coordination, data collection and design of the study. All authors read and approved the final manuscript.
